# Hypoxia triggers IFN-I production in muscle: Implications in dermatomyositis

**DOI:** 10.1038/s41598-017-09309-8

**Published:** 2017-08-17

**Authors:** Noemí De Luna, Xavier Suárez-Calvet, Cinta Lleixà, Jordi Diaz-Manera, Montse Olivé, Isabel Illa, Eduard Gallardo

**Affiliations:** 1 Neuromuscular Diseases Unit, Neurology Department, Hospital de la Santa Creu I Sant Pau, Universitat Autònoma de Barcelona, Institut de Recerca Sant Pau, (Barcelona) and Biomedical Network Research Centre on Rare Diseases (CIBERER), Sant Pau, Spain; 2 Department of Pathology and Neuromuscular Unit, IDIBELL-Hospital Universitari de Bellvitge, Barcelona, Spain; 3 Centro de Investigación Biomédica en Red en Enfermedades Neurodegenerativas (CIBERNED), Barcelona, Spain

## Abstract

Dermatomyositis is an inflammatory myopathy characterized by symmetrical proximal muscle weakness and skin changes. Muscle biopsy hallmarks include perifascicular atrophy, loss of intramuscular capillaries, perivascular and perimysial inflammation and the overexpression of IFN-inducible genes. Among them, the retinoic-acid inducible gene 1 (RIG-I) is specifically overexpressed in perifascicular areas of dermatomyositis muscle. The aim of this work was to study if RIG-I expression may be modulated by hypoxia using an *in vitro* approach. We identified putative hypoxia response elements (HRE) in *RIG-I* regulatory regions and luciferase assays confirmed that *RIG-I* is a new HIF-inducible gene. We observed an increase expression of RIG-I both by Real time PCR and Western blot in hypoxic conditions in human muscle cells. Cell transfection with a constitutive RIG-I expression vector increased levels of phospho-IRF-3, indicating that RIG-I promotes binding of transcription factors to the enhancer sequence of IFN. Moreover, release of IFN-β was observed in hypoxic conditions. Finally, HIF-1α overexpression was confirmed in the muscle biopsies and in some RIG-I positive perifascicular muscle fibres but not in controls. Our results indicate that hypoxia triggers the production of IFN-I *in vitro*, and may contribute to the pathogenesis of DM together with other inflammatory factors.

## Introduction

Dermatomyositis (DM) is an inflammatory myopathy affecting skin and skeletal muscle. The pathogenesis of DM is complex, comprising both immune and non-immune mechanisms. As in other inflammatory myopathies, expression of the major histocompatibility complex class I (MHC-I) is upregulated in the muscle fibres. Muscle biopsy findings in DM show complement deposition in the endothelium, and focal loss of capillaries^1^. In addition, it has been observed that neovascularization has a prominent role in DM^2^ maybe due to a compensatory mechanism. It has been showed that capillary alterations in DM are consistent with whole microvascular units drop out rather than random capillary damage^3^, although a lesion upstream to capillaries has not been proven. However, a correlation between areas of capillary depletion and perifascicular atrophy has been observed^4^.

Tissues with impaired oxygen supply, produced by capillaries loss or clogging, show significant changes in gene expression, mainly modulated by hypoxia-inducible factor 1-alpha (HIF-1α) in a very dynamic process^5^. In normoxic conditions HIF-1α is constituvely hydroxylated. The prolyl hydroxylation represents a posttranslational mechanism to regulate protein interactions. Hydroxylated HIF-1α is ubiquitinated and degradated via proteasome. In contrast, when oxygen is decreased, HIF-1α forms a heterodimer with HIF-1β, that escapes prolyl hydroxylation, and then translocates to the nucleus and binds to hypoxia-response elements (HRE) present in the regulatory regions of hypoxia-inducible genes^6^. These genes are involved in metabolic adaptation (glycogen synthase^7^), angiogenesis (VEGF), and intriguingly, inflammation^8^. Also, it has been shown that inflammatory cytokines (TNF-α and IL1β) can induce HIF-1α expression in rheumatoid synovial fibroblasts^9^.

Microarray studies of muscle biopsies from patients with DM have shown overexpression of IFN-I-inducible transcripts, such as interferon-inducible protein 15 (ISG15), Myxovirus resistance A (MxA), Signal Transducer and Activator of Transcription 1 (STAT1) and retinoic acid-inducible gene I (RIG-I), that were validated at the protein level and specifically detected at perifascicular atrophic areas in DM muscle^10–14^. Interestingly, it has been shown that ISG15 bears HREs in its promoter and a negative feedback loop for HIF-1α in some cell lines has been proposed^15^. RIG-I is an innate immunity receptor of the retinoic acid-like receptors family and an IFN-stimulated gene itself^16^. Activation of RIG-I induces the signalling cascade that involves IRF3 and IRF7 phosphorylation^17^ and consequently IFN-β expression^18^. Since capillary loss is observed in DM muscle we hypothesized that HIF-1α can be stabilized and therefore activate hypoxia inducible genes. Since our *in silico* analysis demonstrated that RIG-I contains HRE in its regulatory regions, we aimed to study if RIG-I expression may be regulated by hypoxia and the effects of that modulation using an *in vitro* approach.

## Results

### Putative hypoxia response elements (HRE) sequences identified in the human *DDX58/RIG-I* and *OAS2* promoters

**Figure 1:**
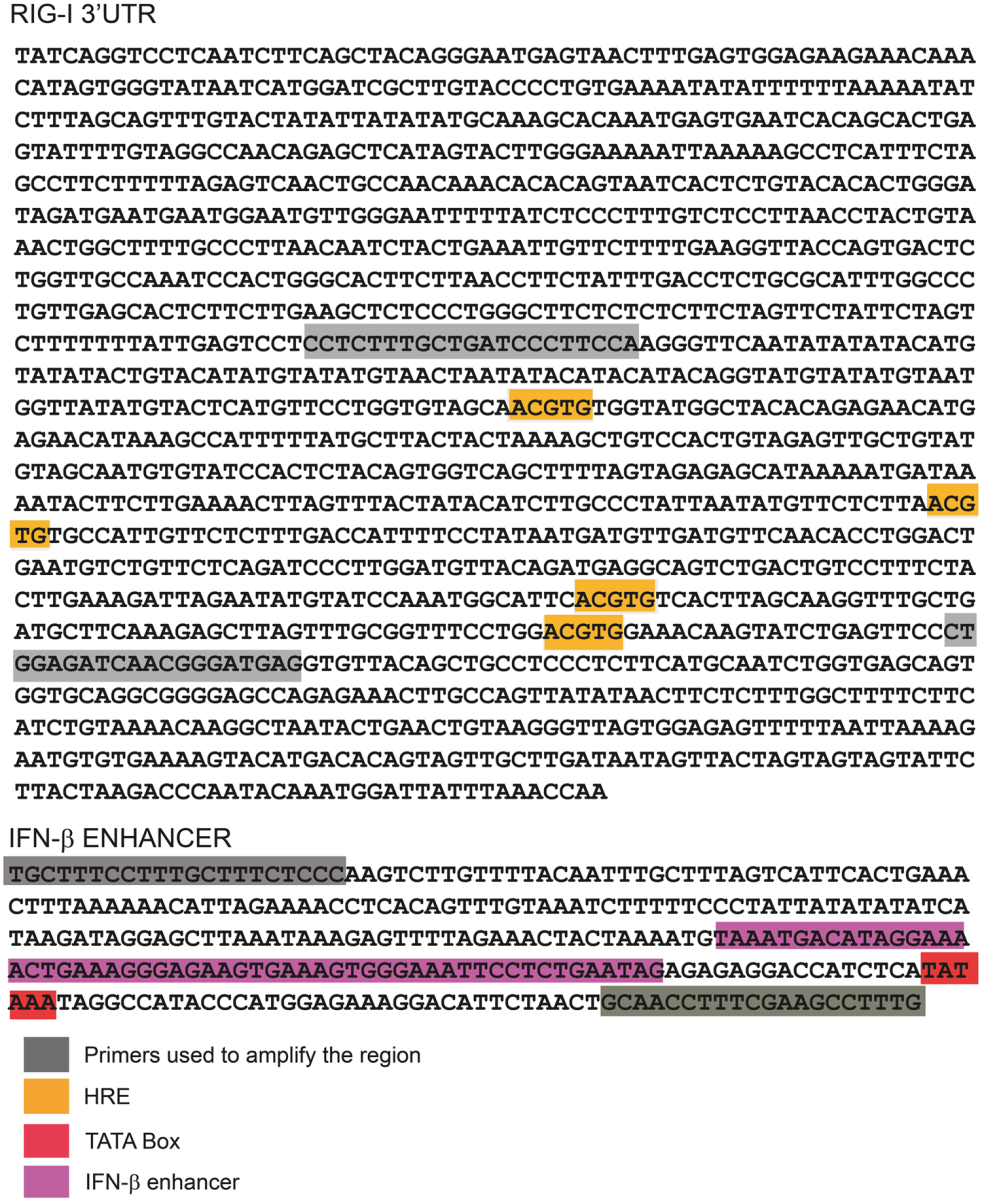
Nucleotides sequences of the cloned fragments of RIG-I 3′UTR and IFN-β enhancer. Sequences of primers used and regulatory elements are highlighted in different colours.

To investigate the possible effect of hypoxia in the transcriptomic induction of genes that belong to the IFN-I signature and previously related with DM^12, 13^, we searched for the presence of HRE in regulatory regions of those genes. Bioinformatic analysis using BLAST alignment tool showed the presence of 4 HRE in the 3′UTR region of *DDX58/RIG-I* (NM_014314.3) (+3470, +3676 +3834, +3892) (Fig. 1). *OAS2* has one HRE in 3′UTR (+2615) (NM_002535 numbered from ATG). In contrast, we did not find any HRE motifs in the regulatory regions of *TLR3*, *RNASEL*, *OAS1*, *OAS3*, β-2-microglobulin a subunit of MHC-I, *IFN-α*, *IFN-β* and *IFN-γ* genes. It has been previously described that *ISG15* has four HREs in 5′UTR^15^.

### 3′UTR region of RIG-I is activated under chemically-induced hypoxia

**Figure 2:**
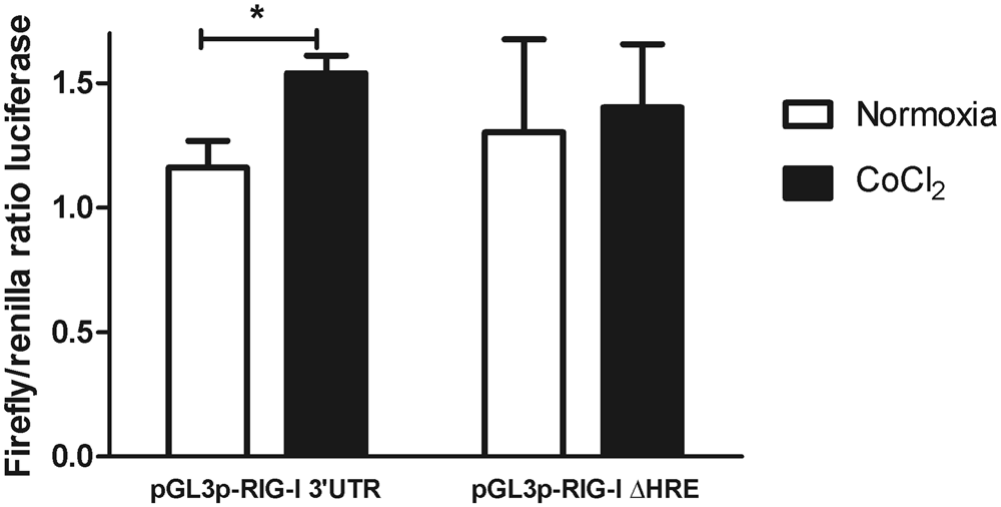
Analysis of the induction of RIG-I transcription by luciferase assays. Transfection of HEK-293 cells with pGL3p-RIG-I 3′UTR cultured in chemically -induced hypoxia (CoCl_2_) induces a significant increase in firefly/renilla luminiscence ratio compared to the same transfected HEK-293 cells cultured in normoxia. No differences were observed when no HREs were present in the plasmid (with pGL3p-RIG-I ΔHRE). Three independent experiments were performed for each condition. Error bars reflect SD. *p < 0.05.

We next addressed the responsiveness of the HRE motifs present in the *RIG-I* 3′UTR region under hypoxic conditions. We performed a functional study using a pGL3p luciferase reporter plasmid including the 580 bp *RIG-I* 3′UTR fragment that contains four HRE motifs. Luciferase assays demonstrated a significant increase in firefly/renilla luminescence ratio under hypoxia compared to normoxia (p < 0.05). We mutated this vector and deleted all HREs (pGL3p + RIG-I 3′UTR ΔHRE) to analyze luciferase activation under hypoxia. We did not find significant differences in the induction of luciferase when no HREs were present in the construct in hypoxia compared to normoxia (Fig. 2).

### Hypoxia induces the expression of *DDX58*/*RIG-I* expression in human myotubes

**Figure 3:**
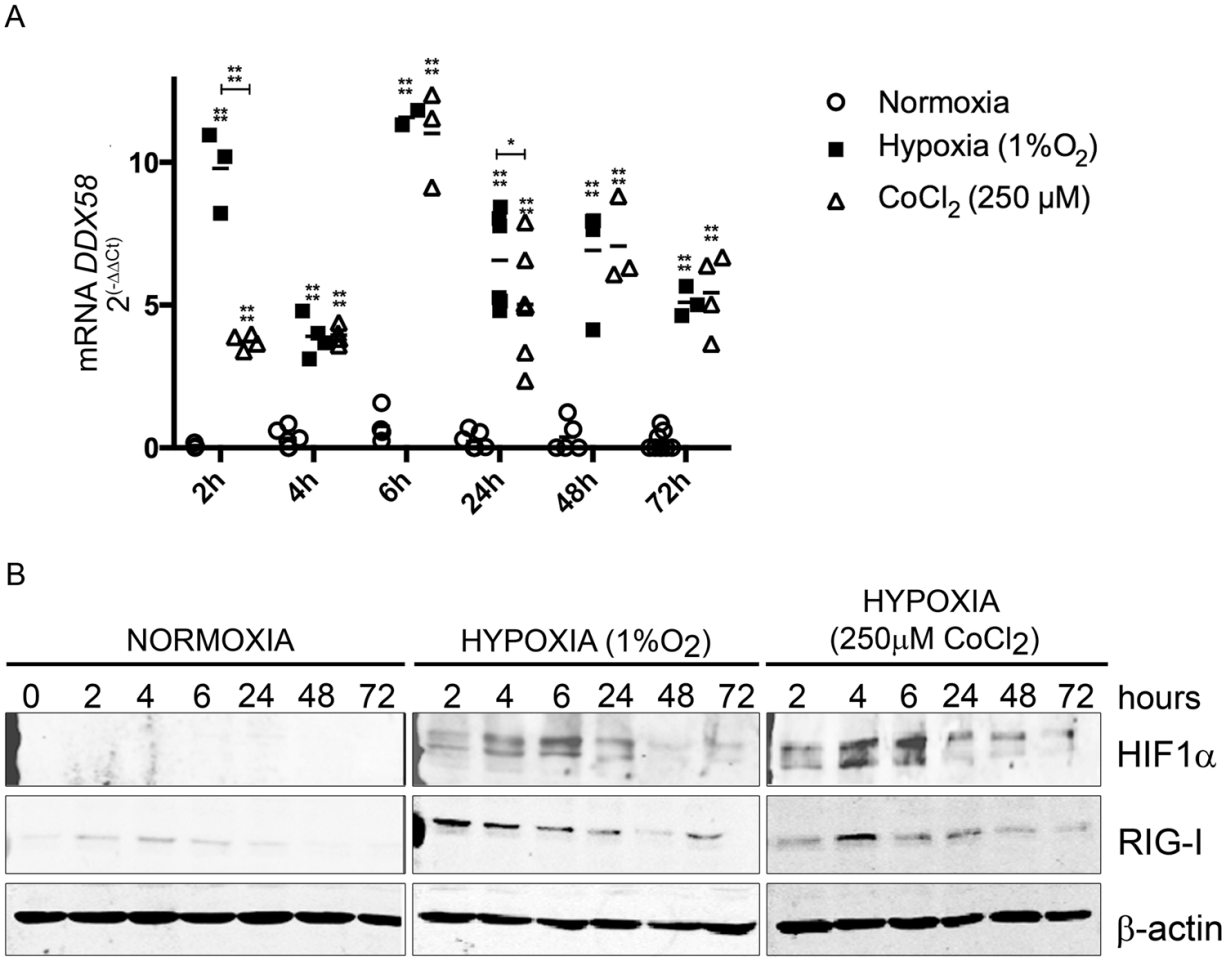
RIG-I is overexpressed in a HIF-dependent manner. (**A**) No *DDX58/RIG-I* mRNA was observed in normoxic conditions. *RIG-I* was increased both in hypoxia (1%O_2_) and in chemically-induced hypoxia (250 μM CoCl_2_). At 2 hours of hypoxia induction, *RIG-I* expression was higher under low levels of O_2_ than after chemically-induced hypoxia. (**B**) RIG-I expression also increases under different hypoxic conditions. Maximum levels of RIG-I were obtained after 2 h with low levels of O_2_. In chemically-induced hypoxia RIG-I overexpression was higher after 4 hours of CoCl_2_ treatment. Three independent experiments were performed. Error bars reflect SD. *p < 0.05; **p < 0.01, ***p < 0.001, ****p < 0.0001.

Our luciferase studies in HEK cells showed that RIG-I transcription is activated under chemically-induced hypoxia with CoCl_2_. We next analyzed if RIG-I expression was induced in human myotubes after both chemically-induced hypoxia (CoCl_2_) and low levels of O_2_. Indeed, a significant increased expression of *RIG-I* mRNA was observed when human myotubes were cultured in a hypoxic chamber at a 1% concentration of O_2_ compared to normoxia. Same results were observed in CoCl_2_ treated cultures (Fig. 3A). In normoxia, levels of *RIG-I* mRNA remained at low levels at all the time points analyzed. These results were confirmed by WB, showing an increased expression of RIG-I when HIF1α expression was increased due to its stabilization (Fig. 3B), either by low levels of O_2_ or by CoCl_2_ induced hypoxia.

### IFNs do not stabilize HIF-1α in human myotubes

**Figure 4:**
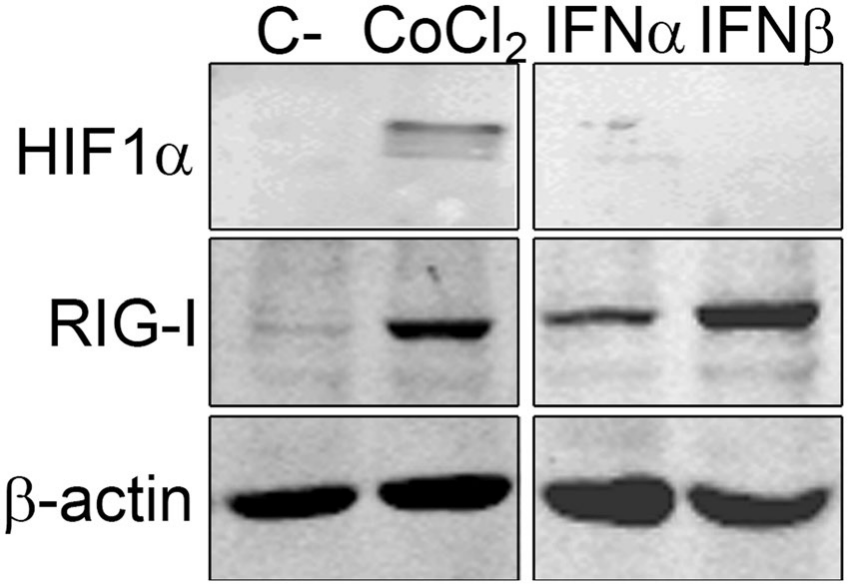
IFN-β stimulation of human myotubes induces RIG-I expression but not HIF-1α stabilization. Human primary myotubes were stimulated with 100 U/ml of IFN-α or IFN-β, during 48 h. Lane1: Myotubes cultured in normoxic conditions; Lane 2: 250 μM CoCl_2_-treated cultures; Lane 3 IFN-α treated cultures; Lane 4 IFN-β treated cultures. Lanes were run in the same gel but were noncontiguous. Three independent experiments were performed.

Our results have shown that RIG-I is a hypoxia-inducible gene in addition to be a known interferon-inducible gene. To exclude the possibility that HIF1α stabilization is directly induced by IFN-I we cultured human primary myotubes with IFN-α or IFN-β and analysed HIF1α expression by WB. No HIF expression was observed 24 and 48 h after stimulation with IFNs. However, we found an upregulation of RIG-I at 24 h and 48 h (Fig. 4) after stimulation with 100 U/ml of either IFN-α or IFN-β. We confirmed the effect of IFNs by analyzing the translocation of STAT-1 to the myonuclei by immunocytochemistry (data not shown). These results indicate that in our cell model the expression of RIG-I can be induced directly by hypoxia independently of IFN-I.

### RIG-I overexpression leads to downstream activation of IFN type I signalling

**Figure 5:**
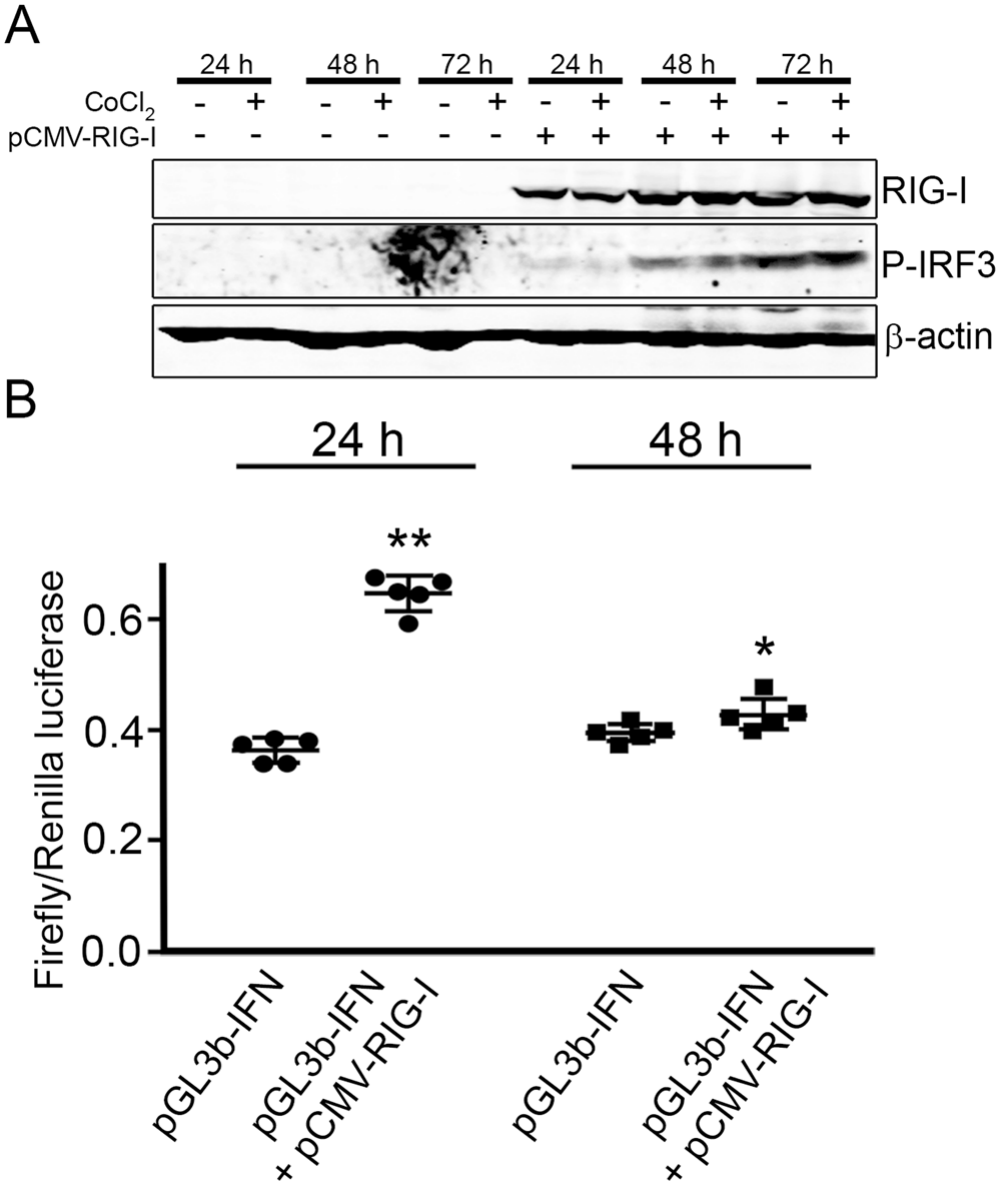
RIG-I overexpression induces IFN-β transcription. (**A**) HEK-293 cells were transfected with a pCMV-RIG-I vector which constitutively expressed RIG-I. RIG-I expression induced phosphorylation of IRF-3, an intermediate effector of IFN-β signalling, both in normoxia and hypoxia at 48 h and 72 h. (**B**) Co-transfection of HEK-293 with pGL3b-IFN-β enhancer and pCMV-RIG-I showed an increased luciferase signal, indicating activation of IFN-β transcription when RIG-I is overexpressed. *p < 0.05; **p < 0.01. Error bars reflect SD. Four independent experiments were performed.

We reproduced *in vitro* the overexpression of RIG-I previously observed in DM biopsies to investigate its functional consequences. We focused on the induction of RIG-I signalling through IRF3 phosphorylation that eventually promotes IFN-I production. Transfection of HEK-293 cells with a vector that leads to constitutive expression of RIG-I (pCMV-RIG-I) increased levels of IRF3-P both in normoxia and hypoxia compared to non-transfected cells (Fig. 5A). Cells transfected with a non-related vector expressing contactin-1 constitutively (pCMV-CNTN1) had no effect on IRF3 phosphorylation. These results suggest that overexpression of RIG-I *per se* induces IFNs signalling pathway and prompted us to hypothesize that high amounts of RIG-I would lead to a higher transcription of IFN-β. To test this hypothesis, we transfected HEK-293 cells with pCMV-RIG-I together with pGL3b-IFN enhancer. Luciferase assay showed an increased of firefly/renilla luciferase ratio (p < 0.001) (Fig. 5B), indicating that RIG-I promotes binding of transcription factors (e.g.IRF3-P) to the consensus sequences in the enhancer of IFN.

### Skeletal muscle cultures under hypoxic conditions release IFNβ

We measured IFN-β release from myotubes cultures at low levels of oxygen (1%) and CoCl_2_ treated cultures compared to those grown in normoxia. Under normoxia no IFNβ was detected in the supernatants at any of the time points analyzed. Under hypoxic conditions, no IFN-β was detected after two hours. However, in CoCl_2_ treated-cultures during 4 h and 24 h, IFN-β concentration was 1.4 ± 0.2 pg/ml and 1.5 ± 0.6 pg/ml. These results were confirmed in muscle cell cultures under 1%O_2_ at the same time points. The concentration of IFN-β in supernatants was 1.5 ± 0.8 pg/ml and 1.9 ± 0.8 pg/ml respectively. No IFN-α was detected in the supernatant of either cultures in normoxia or in hypoxia.

### HIF-1α expression in the muscle biopsies from DM patients

**Figure 6:**
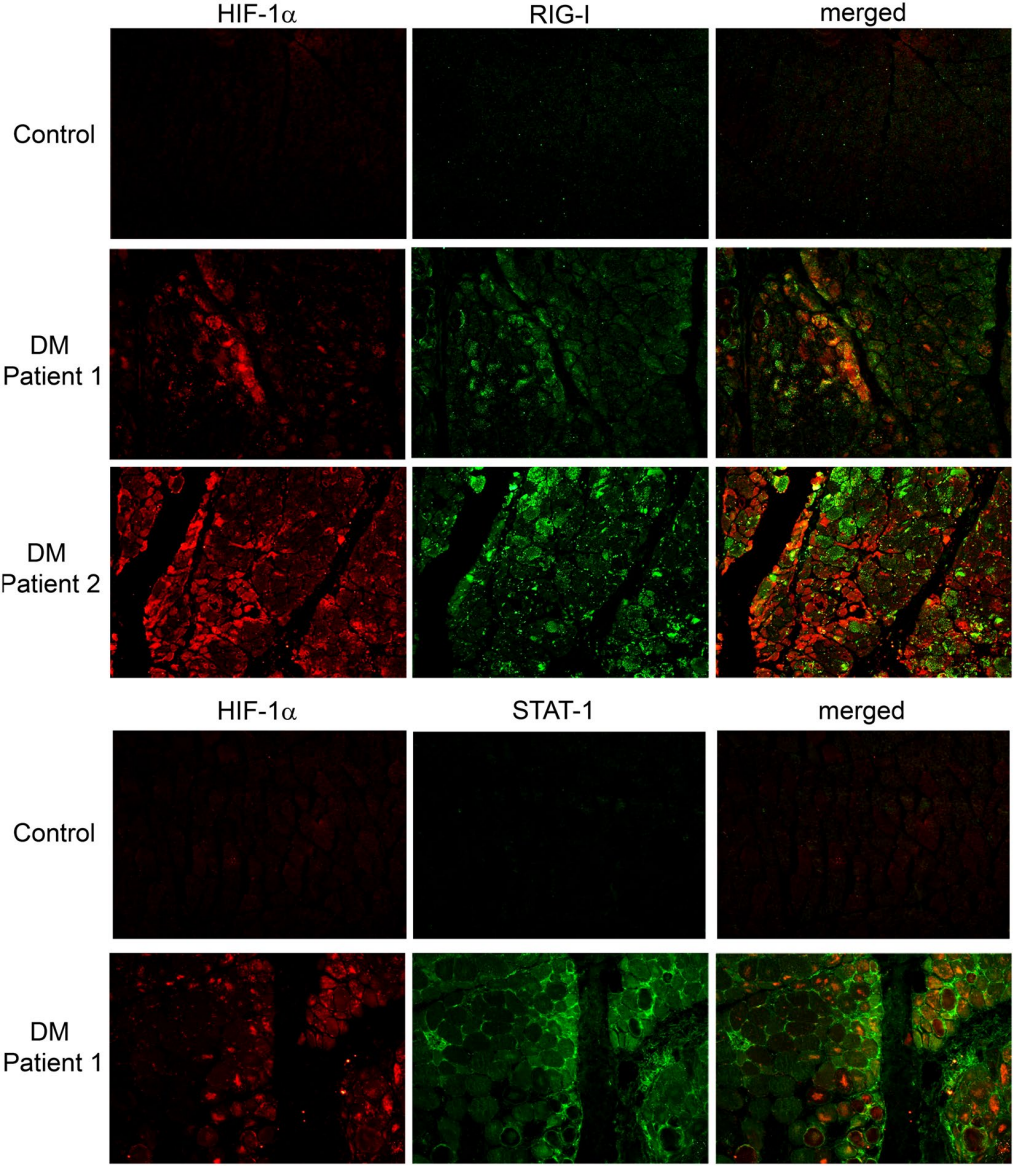
HIF-1α is overexpressed in muscle biopsies from DM patients. Double immunofluorescence show that HIF-1α is present mostly in perifascicular atrophic fibres and some of them are also positive for RIG-I and STAT1. Control muscle biopsies are negative for HIF-1α, RIG-I and STAT1. Magnification ×200.

**Figure 7:**
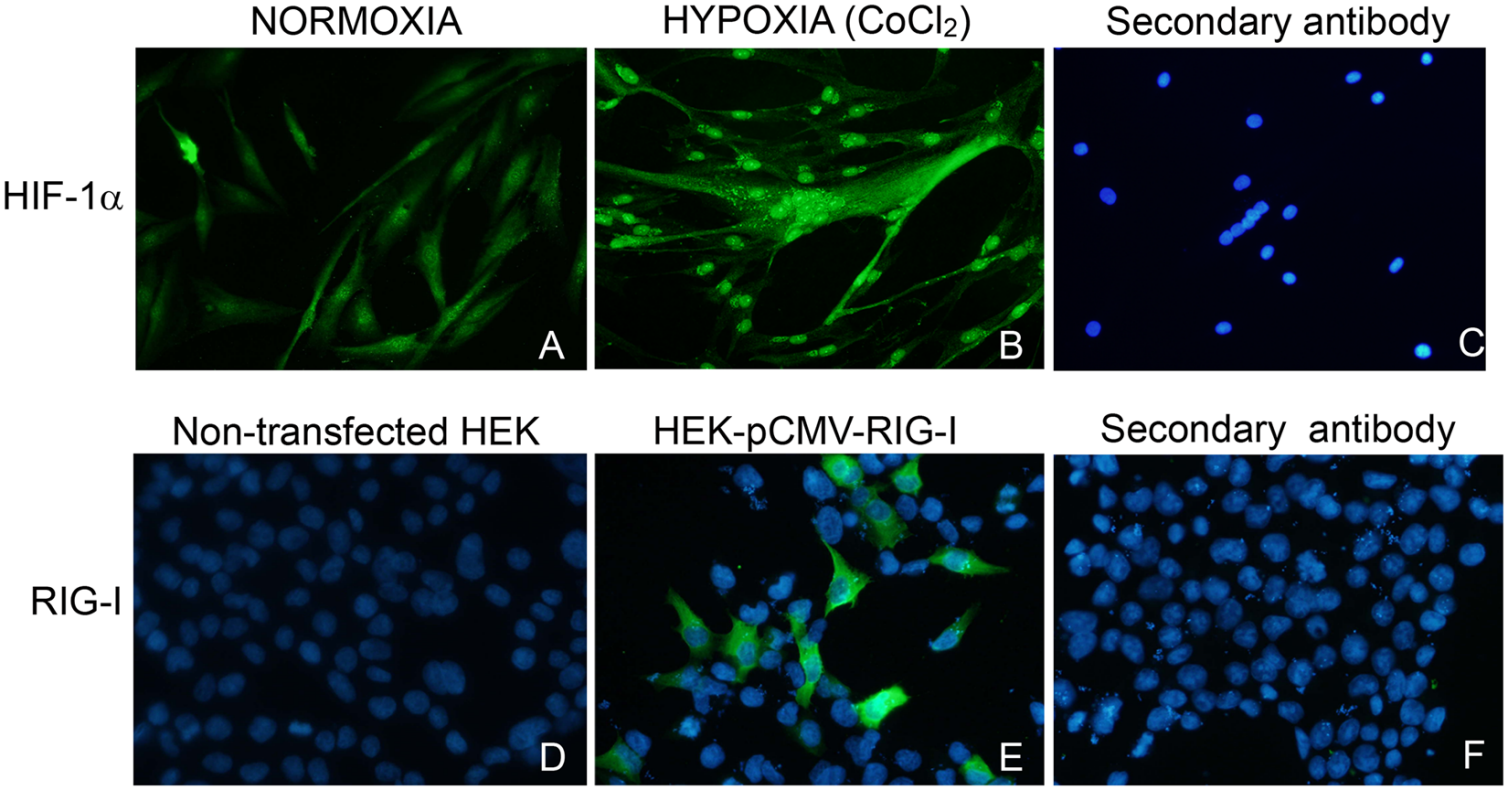
Positive and negative controls for HIF-1α and RIG-I stainings. Human myotubes cultured with CoCl_2_ induce the translocation of HIF-1α to the nucleus. HEK-293 cells were transfected with pCMV-RIG-I. Magnification ×200.

We observed the expression of HIF-1α and RIG-I in all muscle biopsies from DM patients with a similar immunostaining pattern whereas control muscle biopsies were negative (Fig. 6). Although some perifascicular atrophic fibres were positive for both RIG-1 and HIF-1α, we found muscle fibres positive for HIF-1α but not for RIG-I.We also observed HIF-1α positive fibres in intrafascicular areas (Fig. 6). STAT1, another IFN-I signature protein, was also expressed in perifascicular areas that were positive for HIF-1α (Fig. 6). We used muscle cells cultured with CoCl_2_ as a positive control for HIF-1α staining and HEK cells transfected with pCMV-RIG-I as a positive control for RIG-I staining (Fig. 7).

## Discussion

We demonstrate herein that hypoxia induces RIG-I overexpression in human myotubes through HIF-1α stabilization. We show that high levels of RIG-I induce the signalling cascade that activates the transcription of IFN-I and we report the expression of HIF-1α in muscle fibers in DM. We have also shown *in vitro* that human myotubes under hypoxic conditions release IFN-β to the supernatant. We found that the 3′UTR regulatory region of the *RIG-I* promoter contains HREs, and firefly/renilla assays demonstrated that *RIG-I* is a newly described HIF-inducible gene. The experiments in which we removed the HREs present in 3′UTR of *RIG-I* gene confirmed the need of these regulatory regions to induce its transcription under hypoxia.

The expression of RIG-I observed in DM muscle can be induced by IFN and our results demonstrate that hypoxia can also induce RIG-I *in vitro*. We cannot exclude the possibility that in DM, HIF-1α stabilization could be regulated by other factors such as inflammatory cytokines in addition to hypoxia^19^. Although we demonstrated *in vitro* that the overexpression of RIG-I induces the production of IFN-I, it is difficult to establish the sequential activation of secondary messengers in IFN-I signalling pathway *in vivo*.

Although some atrophic fibres were positive for both RIG-I and HIF-1α, we found perifascicular fibres positive for HIF-1α but not for RIG-I. The presence of HIF-1α and RIG-I in the muscle biopsies of DM can be explained by either an inflammatory environment or a hypoxic process. In fact, HIF-1α expression is not confined to perifascicular areas. In the same way, it has been reported that hypoxia inducible proteins accumulate mostly in DM^20, 21^. Expression of STAT1 in perifascicular fibres of DM biopsies is a hallmark of a IFN-I response in this disease^10, 12^. And in fact, we have observed that some perifascicular HIF-1α -positive fibres also express STAT1. We believe that not all STAT1 and/or RIG-I positive fibres are also positive for HIF-1α because the muscle biopsy limits us to analyze the situation at a given time point. HIF-1α has been considered as a transcriptional regulator of immunity and inflammation^22^ and, indeed, some diseases such as rheumatoid arthritis, present high levels of HIF-1^23^. HIF-1α is known to regulate the expression of more than 100 genes that function in a variety of cellular stress responses triggered by low levels of oxygen^22^. This situation may occur in DM muscle where the number of capillaries is compromised. Accordingly, hypoxia enhances the expression of RIG-I in human myotubes. No significant differences in RIG-I expression and IFN-β release were observed between cultures grown in hypoxic environment in a hypoxic chamber and the hypoxia induced in CoCl_2_ treated cultures, which have the advantage of being inexpensive, fast and allows free manipulation of cell cultures without affecting hypoxic conditions^24^.

We did not observe HIF-1α stabilization when we cultured myotubes with IFNs although it has been described in other cellular models^9^. Our results show that the overexpression of RIG-I triggers IRF3 phosphorylation and IFN-β transcription *per se*. Interestingly, it has been reported that RIG-I is also overexpressed in the hippocampus after focal cerebral hypoxia^25^.

In terms of biological significance, pattern recognition receptors must be expressed to control the sterile inflammation response in a hypoxic microenvironment where danger-associated molecular patterns (DAMPs) are released^26^. In heart tissue, it has been reported that extracellular RNA released due to hypoxia and I/R injury activates innate immunity TLR3-Trif signalling and contributes to myocardial inflammation^27^. In animal models of hepatic I/R injury, it has been shown that damage was significantly decreased when the experiments were performed in both, IFN-I receptors and in IRF3-deficient animals^28, 29^. These results support that IFN-I production can be modulated by I/R tissue injury. However in the present study we have not studied the possible release of DAMPs under hypoxia, further studies are needed to demonstrate the presence of DAMPs in DM.

In summary, our results indicate that hypoxia triggers the production of IFN-I *in vitro*, and may contribute to the pathogenesis of DM together with other inflammatory factors.

## Material and Methods

### Patients

All patients (n = 10) were diagnosed of adult DM according to established clinicopathological criteria^30^. For more information, see material and methods section in SI. All muscle biopsies presented perifascicular atrophy and membrane attack complex deposits in vessels, muscle fibres or both. All patients signed an informed consent and the project was approved by the Ethics Committee at Hospital de la Santa Creu i Sant Pau (code IIBSP-MIO-2015-66) in accordance with the Declaration of Helsinki for human research.

### Cell cultures

Control muscle biopsies were obtained from subjects undergoing hip replacement surgery To obtain highly purified myoblasts, primary cultures were sorted for the early surface marker CD56 by immunomagnetic selection. Each 10^7^ cells were mixed with 20 μL CD56-coated microbeads (Milteny Biotec, Bergisch Gladbach, Germany) and incubated at 4 °C for 15 minutes. Unbound microbeads were removed by washing cells in excess PBS buffer followed by centrifugation at 300 × g for 10 minutes. The cell pellet was resuspended in PBS buffer to a concentration of 2 × 10^8^ cells/ mL before separation on a midiMACS cell separator (Milteny Biotec). Only cultures with more than 95% CD56 + myoblasts were used. The purity was tested by immunocytochemistry using anti-CD56 (Becton Dickinson Labware, Franklin Lakes, NJ) or anti-desmin antibodies (Leica, Wetzlar, Germany)^31^.

HEK-293 cell line was purchased from ATCC (Manassas, VA, USA) (CRL-1573) and cultured in DMEM medium supplemented with 5% FBS (Lonza, Basel, Switzerland), 5% of horse serum (Lonza), 1 mM of glutamine (Lonza), 1 mM of sodium pyruvate (Lonza) and antibiotics (Lonza).

Human myotubes were cultured under hypoxia (1%O_2_) in a H35 hypoxystation (Don Whitley Scientific Ltd., West Yorkshire, UK). Chemically-induced hypoxia was performed adding 250 µM of Cobalt (II) chloride hexahydrate (CoCl_2_) to the culture medium (Sigma, St Louis, MO, USA). CoCl_2_ is a stabilizer of HIF-1α that mimics hypoxia responses^32^.

Myotubes were stimulated with 10 and 100 U/ml of IFN-α or IFN-β (Peprotech, Rocky Hill, NJ, USA) in normoxic conditions. Cells cultured in the different conditions were scraped and analyzed by western blot and by Real time PCR at different time points.

### RNA extraction and Real Time PCR

Total RNA from cell samples was extracted using Trizol (Invitrogen, Thermo-Fisher Scientific, Waltham, MA, USA). A 0.5 μg of total RNA was DNAse treated (Invitrogen) and reverse-transcribed into cDNA using the high capacity cDNA RT kit (Life Technologies, Thermo-Fisher Scientific, Waltham, MA, USA). Quantification of the transcript corresponding to *DDX58* (*RIG-I*) (Hs00204833_m1) was performed using TaqMan Universal Master Mix technology (Life Technologies). Desmin was used as internal control (Hs00157258_m1) because its expression was stable in the different experimental conditions. Quantitative PCR was performed in a total reaction volume of 12 μl per well. The comparative Ct method (ΔΔCt) for relative quantification of gene expression was used.

### Western-blot

15 μg of protein were loaded into each lane. Blots were incubated with mAb mouse anti-RIG-I (Novus Biologicals, Littleton, CO, USA), pAb rabbit anti-HIF-1α (Novus Biologicals), rabbit anti-IRF3-P (Abcam, Cambridge, UK) and as a loading control mouse anti-β-actin (Sigma). Dye680 or Dye800 anti-mouse or anti-rabbit secondary antibodies (Li-Cor, Lincoln, NE, USA) were applied (1:7500). Immunoreactive bands were visualized using an Odyssey infrared image system and quantified using Odyssey Software (Li-Cor).

### Immunofluorescence

Immunofluorescence was performed in 10 muscle biopsies of adult DM, in 10 healthy control samples and in cultured cells.

Double immunofluorescence was performed using a monoclonal rabbit anti-HIF-1α (1/25) (Abcam) and a monoclonal mouse anti-RIG-I (1/10) (Novus Biologicals) or anti-HIF-1α and a mouse anti-STAT1 (1/20) (BD Biosciences, Franklin Lakes, NJ). After washing, sections were incubated with biotinylated goat anti-rabbit (Vector Laboratories, Burlingame, CA, USA), followed by streptavidin Alexa-594 (Molecular Probes, Eugene, OR, USA) and goat anti-mouse 488 (1/200) (Molecular Probes). As positive controls we performed HIF-1α staining in human myotubes cultured with CoCl_2_ and RIG-I staining in HEK-293 cells transfected with pCMV-RIG-I. As a negative control, muscle sections and cells were incubated with the secondary antibodies only. Images were obtained using an Olympus BX51 microscope coupled to a DP72 camera.

### IFN-β and IFN-α ELISA

Culture supernatants were concentrated using Amicon Ultra Centrifugal Filters 10 K  (Merck Millipore, Darmstadt, Germany). IFN-β present in the culture media of myotubes exposed to hypoxia (1% of O_2_ and CoCl_2_-treated myotubes) was detected with VeriKine-HS Human IFN Beta Serum ELISA kit (PBL Assay Science, Piscataway Township, NJ), following manufacturer’s instructions. All samples were analyzed by triplicate. The limit of detection was 1.2 pg/ml. IFN-α in culture media was detected with VeriKine-HS Human IFN alpha ELISA kit (PBL Assay Science), the limit of detection was 12.5 pg/ml.

### Construction of Luciferase reporter gene plasmids, directed mutagenesis, transient transfection and luciferase assays

For more details see methods and protocols as SI.

### Statistics

T-test, two-way ANOVA, Tukey’s multiple comparisons test were performed. A value of p < 0.05 was considered significant. GraphPad Prism 5.0 (GraphPad Software, San Diego, CA) was used for data elaboration and statistical analysis.

### Ethical approval

All procedures performed in studies involving human participants were in accordance with the ethical standards of the institutional ethic committee (Hospital de la Santa Creu i Sant Pau,Code No. 12/2009) and with the 1964 Helsinki declaration and its later amendments or comparable ethical standards.

### Informed consent

Informed consent was obtained from all individual participants included in the study.

## Electronic supplementary material

Supplementary methods
