# Supplementary material for: Hypoxia triggers IFN-I production in muscle: Implications in dermatomyositis
Source: Sci Rep. 2017 Aug 17;7:8595. doi: 10.1038/s41598-017-09309-8 (PMC5561123; doi:10.1038/s41598-017-09309-8)
Supplement: Supplementary file 1 — Supplementary methods [file 41598_2017_9309_MOESM1_ESM.doc]

# Supplemental information

**Hypoxia triggers IFN-I production in muscle: Implications in dermatomyositis**

Noemí De Luna^1*^, Xavier Suárez-Calvet^1*^, Cinta Lleixà^1^, Jordi Diaz-Manera^1^, Montse Olivé^2^, Isabel Illa^1+^, Eduard Gallardo^1+^

^1^Neuromuscular Diseases Unit, Neurology Department, Hospital de la Santa Creu i

Sant Pau, Universitat Autònoma de Barcelona, Institut de Recerca Sant Pau,

(Barcelona) and Biomedical Network Research Centre on Rare Diseases (CIBERER), Spain.

^2^Department of Pathology and Neuromuscular Unit, IDIBELL-Hospital Universitari de Bellvitge, Barcelona, Spain. Centro de Investigación Biomédica en Red en Enfermedades Neurodegenerativas (CIBERNED), Spain.

^*^These authors are co-contributing first authors.

^+^These authors are co-corresponding authors.

# Methods and protocols

Patients

Nine patients were female and the mean age was 52 y.o. One patient presented anti-TIF1γ and one anti-Mi2 myositis specific autoantibodies. All patients presented with proximal weakness and the biopsies were taken from biceps (n=3), triceps (n=2), quadriceps (n=3) and deltoid (n=2). Two patients were under treatment shortly before the biopsy was taken. In fact, all muscle biopsies presented upregulation of MHC class I and perymisial and perivascular infiltrates. Age and sex matched control samples (n=10) were obtained from patients who underwent a surgery at the traumatology department but had no weakness or history of neuromuscular disease.

Western Blot

Protein samples corresponding to each cell condition were obtained by scraping, weighing and homogenizing twice for 15 seconds in RIPA buffer containing 10mM Tris/HCl buffer, pH 8, 0.5% sodium deoxycholate, 0.1% SDS, NaCl 140 mM, 1% NP-40. Protein extracts were treated with proteases and phosphatases inhibitors (1:100) (Roche Diagnostics, Basel, Switzerland).

Construction of Luciferase reporter gene plasmids, directed mutagenesis, transient transfection and luciferase assays.

For more details see methods and protocols as SI. We searched for hypoxia response elements (HRE) (A/GCGTG) in the regulatory regions of genes involved in IFN-I signature such as *TLR3*, *RNaseL*, *OAS1*, *OAS3*, *B2M*, *IFN*, RIG-I (*DDX58*), *ISG15* and *OAS2* using BLAST. We used genomic DNA from a healthy volunteer as a template to amplify a 580-bp fragment from +3337 to +3917 RIG-I 3’UTR (NM_014314.3), which contains 4 HRE (A/GCGTG) at +3470, +3676, +3834 and +3892 positions, numbered from the ATG of reference sequence NM_014314. PCR reaction was performed using the following primers: forward primer 5’ CCTCTTTGCTGATCCCTTCCA 3’ that appended a unique KpnI site on the 5’ end and reverse primer 5’ CCTCATCCCCGTTGATCTCCAG 3’ that appended a unique *SmaI* site on the 3’ end.

To clone the enhancer sequence present in the IFN-β promoter (-102 to -46) [20], we designed the following primers: forward primer 5’ TGCTTTCCTTTGCTTTCTCCC 3’ and reverse primer 5’ CAACCTTTCGAAGCCTTTGC 3’ that appended unique HindIII and NheI sites on the 5’ and 3’ ends, respectively. The PCR amplicons were purified using Qiaquick extraction kit (Qiagen, Germantown, MD, USA) and cloned into the p-Spark TA cloning vector (Canvax Biotech, Cordoba, Spain). The p-Spark vector containing the RIG-I 3’UTR amplicon and the pGL3promoter (pGL3p) luciferase vector (Promega, Madison, WI, USA) were digested with *KpnI* and *SmaI* restriction enzymes (New England Biolabs, Ipswich, MA, USA). To clone the *IFN-β* enhancer into the pGL3basic (pGL3b) luciferase vector, the p-Spark vector containing the *IFN-β* enhancer amplicon and pGL3b were digested with *HindIII* and *NheI* (New England Biolabs). This amplicon also contains a TATA box which is a typical core promoter for RNA pol II (Supplemental figure 1). The inserts were ligated into pGL3p or pGL3b vector using T4DNA ligase (New England Biolabs). The correct orientation and sequence of the cloned fragment was confirmed by DNA sequencing, using Big Dye 3.1 (Applied Biosystems, Tehrmo-Fisher Scientific). The pGL3p+3’RIG-I UTR and pGL3b+IFN enhancer plasmids were purified using the endotoxin-free Maxi prep kit (Danagen, Barcelona, Spain).

Directed mutagenesis

The pGL3p+3’RIG-I UTR plasmid was treated with Quickchange Lightning Directed Mutagenesis kit (Agilent Technologies, Santa Clara, CA, USA), to eliminate the HREs from the pGL3p+3’RIG-I UTR plasmid, following manufacturer’s instructions. To delete the first HRE (+3470) the following primers were used: sense primer 5’TGTGTAGCCATACCATGCTACACCAGGAACATGAG3’ and antisense 5’CTCATGTTCCTGGTGTAGCATGGTATGGCTACACA3’. To delete the second HRE (+3676) we used the following primer pairs: sense HRE2 5’AAAGAGAACAATGGCATAAGAGAACATATTAATAGGGCAAGATGTAT 3’ and antisense HRE2 5’ AAAGAGAACAATGGCATAAGAGAACATATTAATAGGGCAAGATGTAT 3’. To delete the third HRE (+3834): sense primer 5’TATGTATCCAAATGGCATTCTCACTTAGCAAGGTTTGCTG 3’, and antisense primer 5’CAGCAAACCTTGCTAAGTGAGAATGCCATTTGGATACATA 3’ and to delete the last HRE4 (+3892) (pGL3p+RIG-I 3’UTR ΔHRE) we used sense primer 5’ CTTAGTTTGTGGTTTCCTGGGAAACAAGTATCTGAGTTCC3’ and antisense 5’ GGAACTCAGATACTTGTTTCCCAGGAAACCACAAACTAAG3’.After cell transfection, we performed mini-preps (Danagen) of 20 colonies to verify the efficiency of the deletion. When we obtained the correct sequence, we performed Maxi Prep kit (Danagen) to obtain high plasmidic DNA concentrations.

Transient transfection

HEK-293 cells were transfected using Fugene HD (Promega), during 48h, following the manufacturer’s instructions. We transfected 0.4 x 10^6^ cells with 2.8 µg of pGL3p+RIG-I 3’UTR or with pGL3p+RIG-I 3’UTR ΔHRE and pGL3b+IFN enhancer DNA. As internal control, we transfected 120 ng of pRLTK (Promega) expressing renilla constitutively under HSV-TK promoter.

We also transfected HEK-293 cells with pCMV-RIG-I plasmid (Origene, Rockville, MD, USA), a RIG-I constitutive expression vector. Non-transfected and pCMV-CNTN1 plasmid (Origene) transfected cells were used as negative controls.

Luciferase assay

We used Dual-Luciferase Reporter Assay System (Promega) to analyze the increment of firefly activity of transfected cells. Transient transfectants were harvested with lysis buffer, and the luciferase and renilla activity were measured with a luminometer (Victor3, Perkin Elmer, Wellesley, MA, USA).
